# Modifiers of mutant huntingtin aggregation: functional conservation of C. elegans-modifiers of polyglutamine aggregation

**DOI:** 10.1371/currents.RRN1255

**Published:** 2011-08-12

**Authors:** Eva Teuling, Annika Bourgonje, Sven Veenje, Karen Thijssen, Jelle de Boer, Joeri van der Velde, Morris Swertz, Ellen Nollen

**Affiliations:** ^*^Department of Genetics, University Medical Centre Groningen, University of Groningen, PO Box 30001, 9700 RB Groningen; ^#^Groningen Bioinformatics Centre, Nijenborgh 7, 9747 AG Groningen, The Netherlands and ^**^Genomics Coordination Center, Department of Genetics, University Medical Center Groningen and Groningen Bioinformatics Center, University of Groningen, P.O. Box 30001, 9700 RB, Groningen, The Netherlands

## Abstract

Protein aggregation is a common hallmark of a number of age-related neurodegenerative diseases, including Alzheimer’s, Parkinson’s, and polyglutamine-expansion disorders such as Huntington’s disease, but how aggregation-prone proteins lead to pathology is not known. Using a genome-wide RNAi screen in a C. elegans-model for polyglutamine aggregation, we previously identified 186 genes that suppress aggregation. Using an RNAi screen for human orthologs of these genes, we here present 26 human genes that suppress aggregation of mutant huntingtin in a human cell line. Among these are genes that have not been previously linked to mutant huntingtin aggregation. They include those encoding eukaryotic translation initiation, elongation and translation factors, and genes that have been previously associated with other neurodegenerative diseases, like the ATP-ase family gene 3-like 2 (AFG3L2) and ubiquitin-like modifier activating enzyme 1 (UBA1). Unravelling the role of these genes will broaden our understanding of the pathogenesis of Huntington’s disease.

## **Introduction**

A number of age-related neurodegenerative disorders, like Huntington’s, Parkinson’s and Alzheimer’s disease, are characterized by the appearance of insoluble protein aggregates in specific brain areas. In Huntington’s disease and other polyglutamine disorders, a genetically-encoded expansion of a polyglutamine stretch in the disease-causing protein renders the mutant protein prone to aggregation [1]. There is a direct inverse correlation between the polyglutamine tract length and the aggregation kinetics, as well as the age of disease onset. In addition, environmental factors and other genetic factors are known to contribute to the disease phenotype [1] [2].

The microscopically visible structures, consisting of disease-specific misfolded proteins, are formed through putative toxic intermediates. While ample evidence suggests that the process of aggregation is coupled to pathology, the toxicity of the different aggregating species remains a matter of debate [3] [4] [5]. Genes that modify protein aggregation might be disease modifiers in Huntington’s disease, in other polyglutamine disorders, or in other related neurodegenerative diseases. Finding these genes will broaden our understanding of the pathogenesis of these neurodegenerative diseases, and may provide novel therapeutic insights.

Small model organisms are especially useful for genetic screening purposes as they are suitable for high-throughput genetic screens. Because their genetic material is highly conserved to humans, such studies can yield results that are relevant for human neurodegenerative diseases as well [6]. A small nematode, *Caenorhabditis elegans*, which transgenically expresses polyglutamine-proteins fused to green fluorescent protein (GFP), shows an age- and polyglutamine-length-dependent aggregation similar to the human disease situation [7]. This model has previously been used for a genome-wide screen in which expression of about 85% of the 17,000 genes of the worm was knocked down by RNAi through feeding [8]. This study revealed 186 genes that, when knocked down, resulted in an increase in aggregation; these genes are therefore potential suppressors of protein aggregation. This set of genes includes a number of genes that function in processes that were already implicated in protein folding and misfolding in different model systems, like a number of molecular chaperones and proteasomal subunits [9] [10] [11] [12]. In addition, genes functioning in processes that were not previously linked to protein aggregation disorders were found, as well as some genes with an unknown function.

In the current study we screened all the human homologs of the modifier genes identified in the *C. elegans­*-screen [8]. Several studies have already confirmed the validity of *C. elegans-*genetic screens to study human protein aggregation disorders, by assessing the effect of knockdown of the homolog genes on protein aggregation in other human cell models [13] [14] [15]. Here, we investigated the role of orthologs of all the modifiers of polyglutamine aggregation found in *C. elegans* in mutant huntingtin aggregation.

In human cultured cells, huntingtin aggregation can be modelled by using expression constructs containing the first 67 amino acids of the huntingtin protein with an internal stretch with a variable number of glutamines fused to GFP [16]. In this study, we expressed Htt-Q74-GFP and specifically knocked down the human orthologs of the *C. elegans*-genes by using shRNA constructs. We show that 26 human orthologs of the 186 *C* *. elegans*-modifier genes from our previous study have a robust effect on the aggregation of mutant huntingtin. Among these are genes which already have confirmed roles in mutant huntingtin aggregation, validating our experimental approach. In addition, we identified a number of genes with no previously known involvement in the aggregation of mutant huntingtin, such as the eukaryotic translation initiation, elongation and termination factors.

## **Materials and methods****

### *Orthology of C. elegans genes*

To determine the human homologs of the 186 *C* *. elegans* genes identified in the genome-wide RNAi-screen, the KEGG-database was accessed through the KEGG API by a program script (source code available at http://www.molgenis.org/browser/molgenis_apps/trunk/apps/xgap/miscellaneous/kegg/Run.java?rev=8265). It attempts to map any gene identifier to the corresponding KEGG entry for the gene, followed by finding its closest orthologous gene in a specified target organism. The closest (or 'best match') ortholog was found by using a combination of the 'KEGG Orthology' and 'KEGG Sequence Similarity Database' sources. Homology was confirmed by reverse orthology (e.g. searching for the *C. elegans* ortholog of the human gene); some genes were discarded if they: (1) did not have a human ortholog, (2) showed reverse orthology to another *C. elegans* gene, (3) had homology with more than one *C. elegans* gene, or (4) the homology-score was lower than 0.20. In total, 177 human homologs were identified, the identity between human and *C. elegans* genes was calculated on the amino-acid level. 

### Design siRNA-targets and shRNA-oligonucleotides and cloning strategy

Three siRNA-targets per gene were designed through the OligoEngine website using the Gene Silencing RNAi Design Tool optimized for pSUPER vectors. Target sequences were designed on the cDNA-sequences as found in the NCBI database. Three 19-nucleotide target sequences were selected based on the ‘general rules’ of RNAi-design [17] [18], the ‘recommended targets’ selected by the program, by the number of Blast hits, and by the potency and distribution of the target regions over the mRNA-sequence. For some genes, especially small ones, only one or two siRNA-sequences could be designed. The 19-nucleotide-sequences were used in a standard pSUPER-cloning strategy [19]. 

The pSUPER-vector was digested with BglII and HindIII overnight at 37ºC. 60 base-long oligonucleotide sequences were ordered in 96-well plate format at a concentration of 50µg/µl. For annealing, forward and reverse oligos were combined with milliQ and NEB buffer 2, heated for 15 min to 95ºC and cooled overnight. The annealed sequences were diluted 1:50 in milliQ, and 1 µl was used in an overnight ligation at 16ºC with 50 ng digested vector. 5 µl of the ligation mix was transformed to 25 µl competent DH5α by heat-shock and plated on 6 cm LB-Amp-plates. Three colonies per ligation were used for colony-PCR with primers pRS for (CCCTTGAACCTCCTCGTTCGACC) and pRSrev (GAGACGTGCTACTTCCATTTGTC). Positive clones showed a 650 bp-insert, compared to negative clones showing a 600 bp-insert, this difference was observed on a 1.5% agarose gel. Positive clones were grown in liquid cultures to obtain plasmid DNA and sequences were verified by sequencing with pRSseq (GCTGACGTCATCAACCCGCT). 

## Cell culture and transfection

HEK293-cells were cultured using standard conditions (DMEM+10%FBS+1% penstrep). For transfection, 15*10^5^ cells were plated into 12-well plates on glass coverslips coated with poly-D-lysine. After 16 hours, cells were transfected with equal amounts of shRNA-vectors and Htt-exon1-Q74-GFP (16) using PEI as the transfection agent (a total of 1 µg DNA per well). After 48 hours, cells were fixed with 4% PFA for 20 minutes at room temperature, followed by washing with milliQ and dehydration in 70% and 100% alcohol, and mounted on glass slides using Vectashield containing DAPI. In every experiment, shRNA-constructs with a scrambled (SCR) 19nt-sequence, which did not target any part of the human genome, shRNA targeting green fluorescent protein (GFP), and shRNA targeting chaperonin-containing TCP1, Subunit 2 (CCT2) were used as positive controls. 

## Aggregate counting and selecting positive candidates

Aggregates were counted using two different techniques. One technique was making multiple images of the GFP-signal and the DAPI-staining per coverslip by regular fluorescence microscopy. Using a batch macro made in ImageJ, pictures were analyzed to count the number of cells and the number of aggregates. The number of aggregates was calculated as percentage aggregates per total number of cells, averaged over 5 images. Coverslips were also photographed using TissueFAXS (TissueGnostics, Vienna, Austria) and DAPI-staining, and aggregates were monitored using TissueQuest software. Comparing ImageJ/TissueQuest results of the same experiment showed similar results. 

To be able to compare the results of different experiments, the level of aggregation with the SCR shRNA was set to 100%; the results of the knockdown experiments in the same experiment were normalized to this number. When shRNA-constructs targeting GFP or CCT2 did not lead to a reduction or induction of aggregation, the experiment was repeated. After the initial screen, positive shRNA-constructs were identified if they showed an induction of the percentage of aggregates of at least 150% compared to the SCR-shRNA. The experiments with these shRNA-constructs were repeated in triplicate. 

Figure 1 presents a graphic overview of the experiments.  

**Figure:**
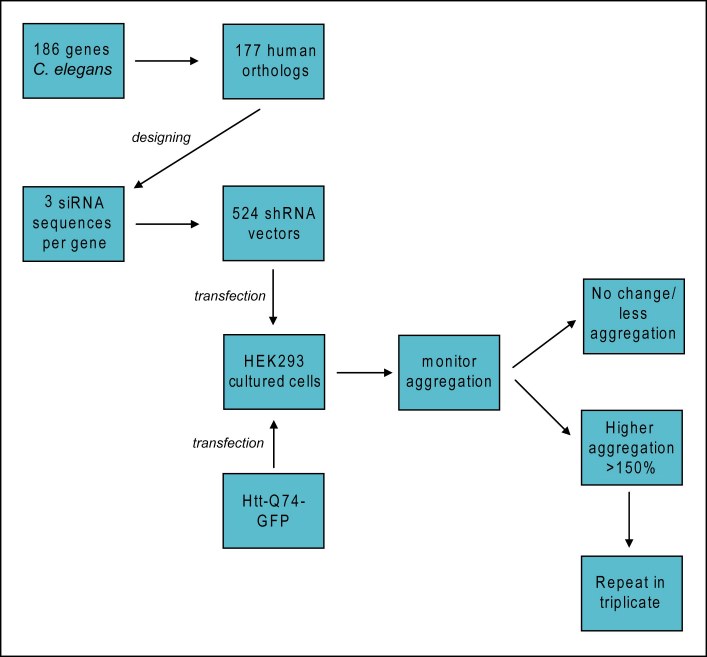
*Figure 1. Flowchart of experimental procedures. Experiments performed to test 186 C. elegans suppressors of polyglutamine aggregation for their conserved function on mutant huntingtin aggregation in mammalian cells. See text for further explanations and selection criteria. *

## **Results**

### *C. elegans* *modifiers of protein aggregation are highly conserved to humans*

In a *C. elegans *screen for modifiers of polyglutamine aggregation, we previously identified 186 *C* *. elegans* genes that modify polyglutamine aggregation [8]. To establish functional conservation of the human counterparts of these genes, we identified the closest human orthologs of these genes by automated analyses. The KEGG database retrieval script provided us with information about BLAST identity, gene name, definition, NCBI-GI, GeneID, GenbankID, and amino acid and nucleotide sequences. A reverse test was performed by searching the *C. elegans* homolog of the human gene to avoid paralogy. 

 In total, we found 177 human genes that had at least 20% amino acid sequence homology to a human gene (listed in table 1). 70% (124/177) of these human genes had more than 40% identity on the amino acid level with their *C. elegans* homolog (table 1). High identity was found within the groups of protein degradation, folding, synthesis and transport (mean identity 60%, of which 85% of the genes had an identity >40%). These are essential cellular processes that are highly conserved throughout evolution. For nine of the *C. elegans* genes, no human homolog could be assigned for various reasons: for five genes, there was another gene in the list that led to the same human homolog (these were doubles); three genes were not conserved to humans at all, and one gene no longer existed in the databases searched.

Table 1. Human orthologs of *C. elegans* modifiers of polyglutamine aggregation and their effect on mutant Huntingin aggregation in cultured mammalian cells

**Figure:**
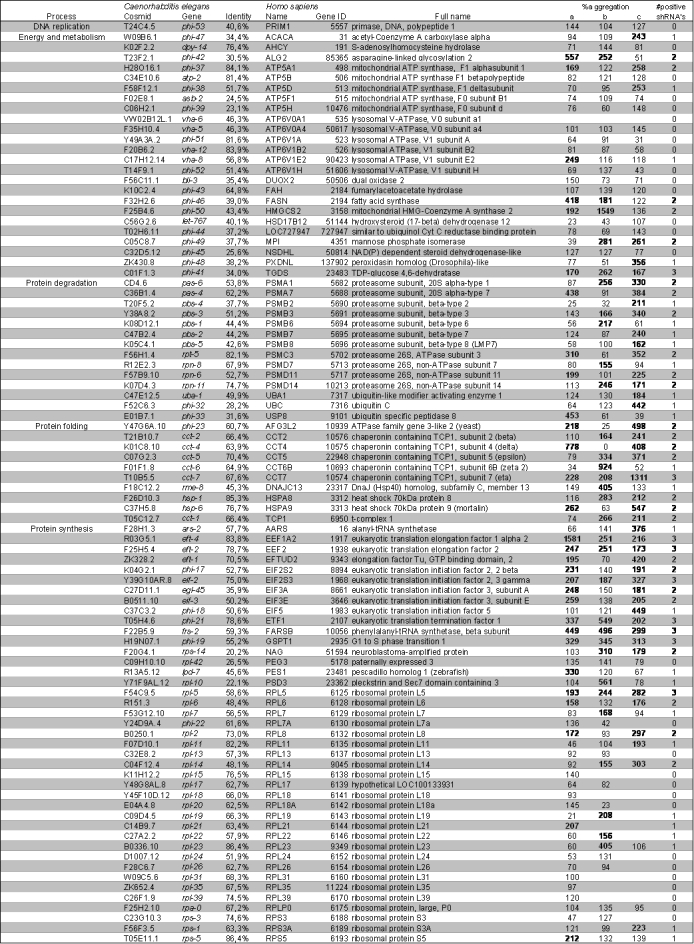

**Figure:**
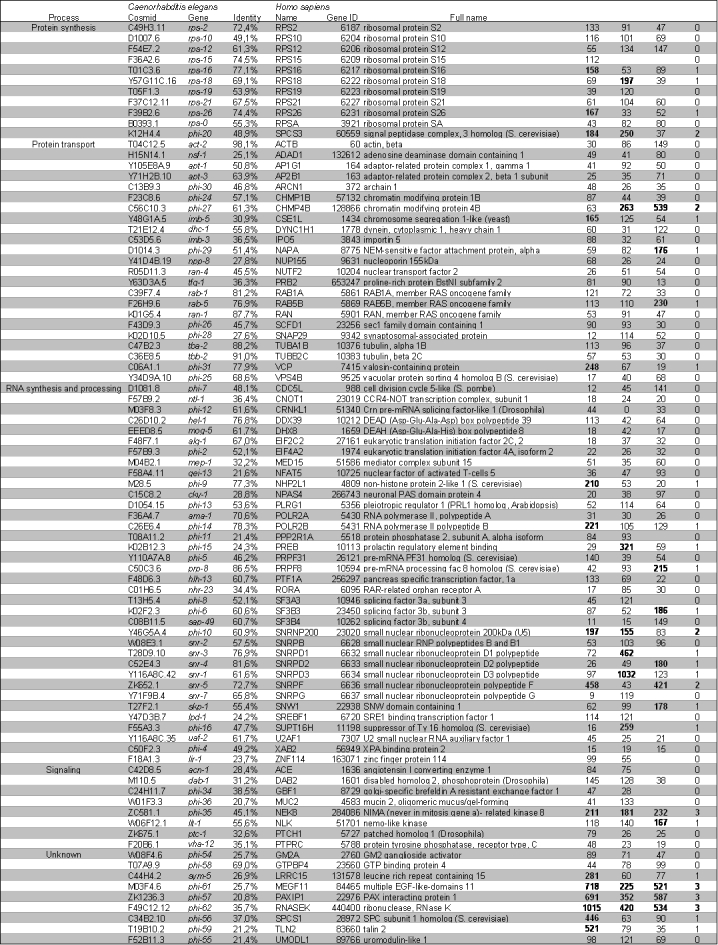
*Genes are grouped in functional classes as previously assigned (8). C. elegans cosmid and gene, human gene name, Gene ID, and full name are shown, as well as their identity on the amino acid level. Columns a, b and c represent different individual shRNA-constructs and the percentage of mutant huntingtin aggregation in that experiment compared to the scrambled control. The number of shRNA-constructs with an aggregation induction >150% for that gene is given in the last column. *

*shRNA-mediated knockdown of 90 genes in cultured cells expressing Htt-Q74-GFP led to an increase in Huntingtin aggregation*

To establish the role of these genes in the aggregation of mutant huntingtin (Htt) in human cultured cells, we generated three shRNA-constructs per gene and transfected these in combination with a huntingtin-exon1 construct with 74 glutamines, fused to GFP (Htt-Q74-GFP) to HEK293-cells. We measured the number of aggregates per total number of cells. As a negative control, we used an SCR sequence that did not target any human genes as verified by BLAST. In addition, shRNA-constructs targeting the DNA sequence of GFP were used as positive controls; these shRNA-constructs resulted in a reduction of protein aggregation (figure 2).

In the original screen in *C. elegans, *six members of the chaperonin TRiC were found to be modifiers of polyglutamine aggregation [8]. Earlier studies have confirmed the role of these genes in the aggregation of polyglutamine and mutant huntingtin in yeast [20], neuron-like cells [14], and cultured human cell-lines [13], including HEK-293 cells. To test the validity of our screen, we first designed shRNA-constructs against the human homologs of these genes and observed that knockdown of the majority of these subunits led to an increase in mutant huntingtin aggregation (table 1, figure 2).

**Figure:**
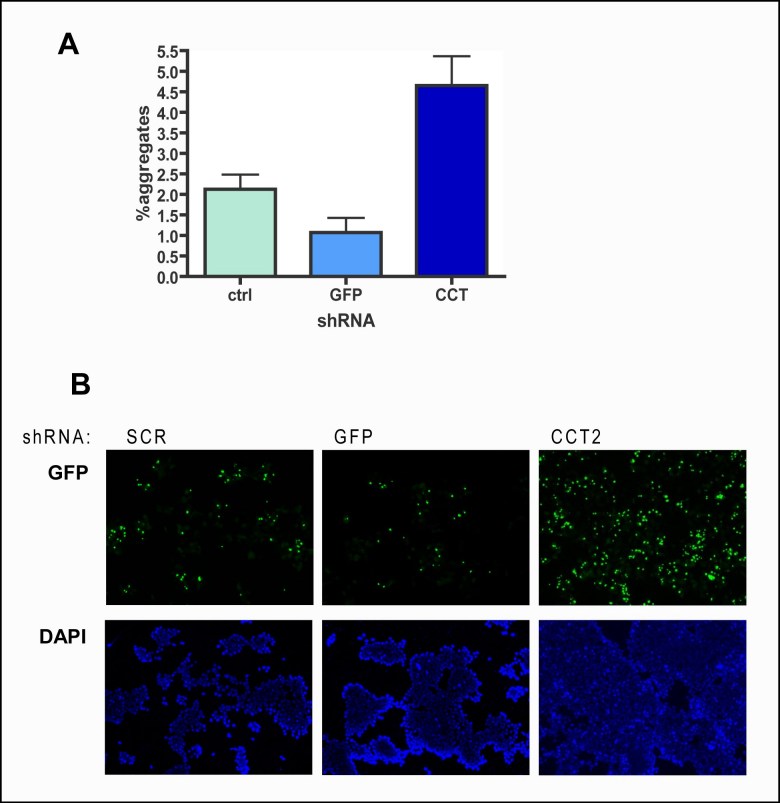
*Figure 2. Validation of experimental procedures **(A)** Percentage of cells with Htt-Q74-GFP aggregates using control shRNA-constructs and vectors targeting GFP and CCT2. The error bar represents the standard error of the mean (SEM) of 10 individual experiments. **(B)** Representative fluorescent images from these experiments with green channel showing aggregates (top row) and blue channel showing nuclei as stained by DAPI (bottom row).*

We evaluated alterations of aggregation of mutant huntingtin in cells transfected with a total of 524 shRNA-vectors (table 1). The cut-off for induction of aggregation was set at 150% compared to the SCR shRNA within the same experiment. Of the original 524 shRNA-constructs corresponding to the 177 genes, 147 shRNA-constructs, representing 90 different genes, induced aggregation of more than 150% (table 1).

Knockdown of a number of genes showed an opposite effect compared to their effect on polyglutamine aggregation in *C. elegans*. For example, knockdown of many ribosomal subunits led to lower aggregation levels. This was also the case for some ATPases and ATP-synthases. Further, knockdown of the genes previously predicted to be involved in protein transport did not have an obvious effect on the aggregation of mutant huntingtin (table 1). 

In sum, not all the genes identified as suppressors of polyglutamine aggregation in *C. elegans* also function as suppressors of mutant huntingtin aggregation in cultured mammalian cells. 

### 26 human genes were validated as suppressors of mutant Huntingtin aggregation

In the first set of experiments, for 30 genes two shRNA-constructs led to an induction of mutant huntingtin aggregation of >150%, and for 13 genes this was the case with all three shRNA-constructs (see also table 1). Thereafter, experiments with shRNA-constructs for these 43 genes were repeated in triplicate, for all three shRNA-constructs. The results were averaged and after three repeats, 58 shRNA-constructs, corresponding to 26 human genes, showed an induction of mutant huntingtin aggregation of >150%. The percentage of aggregation of the three repeats and the averages can be found in figure 3 and table 2.

**Figure:**
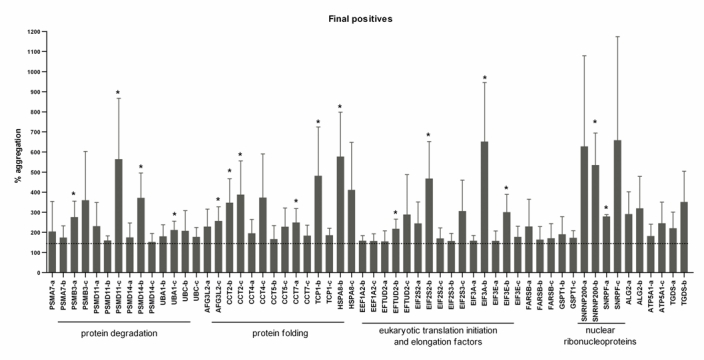
Figure 3. Mutant huntingtin-aggregation induction by 58 different shRNA-constructs representing 26 individual genes. Average induction of aggregation versus control of final positives after three repeats. Only the genes where >2 shRNA-constructs showed induction of aggregation are shown. Bars represent the standard error of the mean (SEM) of the three experiments. * = p-value <0.10 as determined by z-score.

It can be seen that several functional classes are overrepresented among the final positives. Some of these cellular pathways, like protein folding and degradation, play a role in protein quality control and are therefore obvious regulators of protein aggregation. For example, two or three shRNA-constructs targeting the genes for four of the eleven proteasomal subunits in the *C. elegans* screen (*PSMA7, PSMB3, PSMD11 and PSMD14*), led to an induction of mutant huntingtin aggregation of >150%. And of the six CCT subunits in the *C. elegans*-screen, five (*CCT2, CCT4, CCT5, CCT7 *and* TCP-1*) met the criteria for being a suppressor of mutant huntingtin aggregation in this study. In our *C. elegans* screen, two hsp70-chapersones (homologs of *HSPA8* and *HSPA9*) were found to be modifiers of aggregation, in the human cells only *HSPA8* was a modifier of huntingtin aggregation. However, one shRNA for *HSPA9* also induces aggregation to 140%. 

shRNA-constructs targeting four eukaryotic translation initiation factors and two eukaryotic translation elongation factors also led to an induction of mutant huntingtin aggregation. Their role in protein aggregation, or in mutant huntingtin aggregation more specifically, is novel. 

Furthermore, shRNA-constructs targeting two nuclear ribonucleoproteins, *SNRNP200* and *SNRPF*, led to an induction of mutant huntingtin aggregation. The proteins encoded by these genes function in a complex with the SMN protein; mutations in the *SMN* gene are the causative factor in spinal muscular atrophy, implicating a general role for the protein in neurodegeneration [21]. 

Table 2. List of genes in which knocking down by two or three shRNA-constructs leads to an induction in mutant huntingtin aggregation

**Figure:**
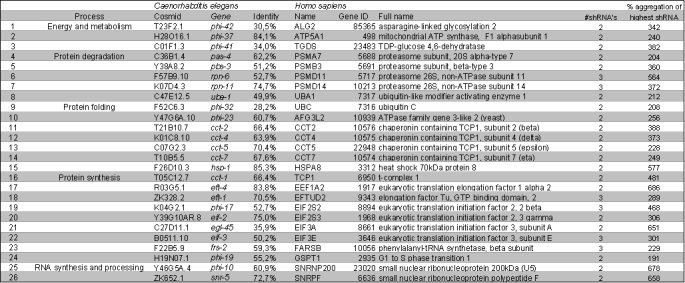
*Functional classes as previously assigned (8), C. elegans cosmid and gene, human gene name, Gene ID, full name and identity are shown, as in table 1. #shRNAs represent the number of shRNA-constructs that lead to an increase in aggregation of >150% after three experiments. The last column shows the highest average induction observed for that gene*

In sum, in this study we have shown that 26 modifiers of polyglutamine aggregation in *C. elegans* have a conserved function towards modifying mutant huntingtin aggregation in cultured human cells and we identified some genes not previously implicated in this pathway.

## Discussion

### Previously identified modifiers of (huntingtin) aggregation 

In the current study, we have monitored the effect on protein aggregation of *C. elegans* genes shown to modify polyglutamine aggregation, in a human cell line [8]. The *C. elegans* screen was focused on finding genes that, upon knockdown, increase aggregation and are therefore *suppressors* of protein aggregation. The original screen in *C. elegans* revealed the aggregation-suppressing function of some genes that were previously linked to protein aggregation, including a number of chaperonins [8] known to assist in protein folding. Earlier studies had already confirmed their functional conservation towards modifying mutant huntingtin aggregation in human cultured cells [13] [14] [15] and in yeast [20]. In the study by Kitamura *et al*, [13] knockdown of CCTζ (CCT6A) and CCTα (TCP-1) led to stimulating aggregation of expanded polyglutamine and mutant huntingtin in HEK293- and HeLa cells, respectively. Tam *et al* [14] reported knockdown of subunits of the Chaperonin-Containing TCP1 Complex (CCT), which is believed to reduce total CCT-levels, leading to higher levels of aggregation in N2A-cells. This is in line with our results, which show that five out of the six chaperonin-subunits found in the *C. elegans* screen are suppressors of mutant Huntingtin aggregation (table 2).

Of the eleven proteasomal subunits in the *C. elegans* screen, four were also confirmed as aggregation-modifying genes in our study. It is well known that the proteasome is involved in the targeting and degradation of misfolded proteins, but the exact role of the proteasome in the degradation of expanded polyglutamine proteins remains debated [22] [23]. Inclusions in neurons from Huntington disease models can be labelled with antibodies against ubiquitin [24] [25], but it is unclear whether the proteasome can degrade expanded polyglutamine proteins [26] [27]. Similar conflicting results have been obtained when proteasomal activity in brain was measured – global activity does not seem to be altered but there may be changes on the cellular/subcellular level [28] [29] [30]. Chemical reduction of proteasome activity leads to an increase in the amount in huntingtin fragment levels [22] and, in addition, proteasome activity has been show to decline with age, consistent with the late onset of Huntington’s disease [23]. This is in line with our study, in which we observed an increase in mutant huntingtin aggregation upon knockdown of four proteasomal subunits. 

We identified ubiquitin-like modifier activating enzyme 1 (UBA1) and ubiquitin C (UBC) as modifiers of mutant huntingtin aggregation. Variations in the ubiquitin system have been associated with Huntington’s disease and other neurodegenerative diseases [31]. More specifically, mutations in the UBA1-gene (or UBE1) have been associated with X-linked infantile spinal muscular atrophy [32], a neurodegenerative disease of the motor neurons. Another link between ubiquitin-genes and Huntington’s disease comes from heterozygous *Ubc* knockout mice: when these animals were crossed with a Huntington’s disease mouse model, there was an improvement of the disease phenotype [33]. This would suggest that knockdown of UBC leading to an increase in mutant huntingtin aggregation may be beneficial to the cells, but our study demonstrates the opposite. However, genes involved in protein folding, degradation and ubiquitination, which are linked to protein aggregation in general, are also modifiers of mutant Huntingtin aggregation in cultured mammalian cells.  

### Novel modifiers of huntingtin aggregation

Our study also showed the involvement of a number of genes in mutant Huntingtin aggregation that were not previously linked to protein aggregation. One of these genes is the ATPase family gene 3-like 2 protein (AFG3L2). No role for this gene has been reported so far in mutant huntingtin aggregation, but mutations in AFG3L2 cause the dominant spinocerebellar ataxia, SCA28 [34], a neurodegenerative disorder characterized by Purkinje cell degeneration. This gene is closely related to paraplegin, which, when mutated, causes an autosomal recessive form of hereditary spastic paraplegia (HSP) [35]. AFG3L2 and paraplegin together form the mitochondrial AAA-protease, its yeast homologs function in mitochondrial protein quality control [36] [37]. Altered assembly of the protease-components is the pathological feature in HSP. Lower expression levels of subunits of this system are mimicked in our experiments by shRNA-constructs. This might lead to an altered assembly of the protease, causing aberrations in the protein quality control system in general, which leads to higher aggregation levels.

A major group of modifiers of mutant huntingtin aggregation are the eukaryotic translation initiation and elongation factors. Knockdown of *EEF1A2, EFTUD2, EIF2S2, EIF2S3, EIF3A* and *EIF3E* led to induction in mutant huntingtin aggregation in our screen. More subunits of this complex were found in the *C. elegans* screen as modifier genes, but just did not reach >150% aggregation induction, indicating that they might induce aggregation on lower levels. Subunits of this complex, like *EIF2*-alpha, have been implicated in autophagy and protein aggregation. For example, heat shock protein HspB8 induces the phosphorylation of the alpha-subunit of eiF2, which is involved in the suppression of aggregation through autophagy [38] [39]. In addition, altered levels of eiF-proteins are found in degenerating brain regions of Alzheimer’s patients [40], further implicating a function for these proteins in stress response. So, similar to lower expression of heat shock proteins, shRNA-constructs targeting eiF-subunits could also reduce the autophagic response of cells to different stressors and thereby cause higher levels of protein aggregation.  

In addition, knockdown of *SNRP200* and *SNRPF*, encoding two small nuclear ribonucleo­proteins (snRNPs), led to an induction in mutant huntingtin aggregation in the present study. snRNPs have been implicated in neurodegenerative disorders before: for example, snRNPs are in a complex with the SMN protein [21]. Furthermore, a mutation in *SNRP200* causes autosomal dominant retinitis pigmentosa [41], a degenerative disorder of the retina. Our results imply that there might be a relationship between SMA and autosomal dominant retinitis pigmentosa and protein aggregation. 

Another interesting new candidate gene for modifying protein aggregation is *FARSB* (phenylalanine-t-RNA synthetase, beta-subunit). All three shRNA-constructs targeting this gene led to a induction of mutant huntingtin aggregation of >150%. It was previously shown that mutations in another tRNA-synthetase in mice (alanyl-tRNA synthetase, Aars) led to protein misfolding and neurodegeneration[42]. In this study it was also demonstrated that low levels of mischarged tRNAs already lead to an accumulation of misfolded proteins in neurons, indicating that general alterations in this pathway might be deleterious to these cells. The human homolog of this gene, AARS, was present in our screen, but only one shRNA-construct led to an induction of protein aggregation of >150%, so it was excluded after the first screening round. 

It is interesting to note that many novel modifiers of huntingtin aggregation, as identified in this study, function in RNA-processing and translation pathways, (e.g. translation initiation and termination factors, some SNRNPs and FARSB). This is in line with recent evidence that mRNA can play a pathogenic role in neurodegenerative disorders: for example, spinal muscular atrophy (see above) [21] is caused by a mutation in the SMN gene that is involved in the formation of a complex functioning in RNA-splicing. Recently identified mutations in TDP-43 and FUS, both RNA-binding proteins, have been shown to cause the neuro­degenerative disorder amyotrophic lateral sclerosis (ALS). Myotonic dystrophies (DM1 and DM2) are caused by repeat expansions in RNA, while Fragile X syndrome (FXTAS) is caused by a repeat expansion in the 5’UTR of the FMR-1 gene. Some recent evidence for RNA toxicity in the polyglutamine disorders has also been published, as reviewed in [43]. Thus our screen confirms earlier assumptions that modifications in RNA-processing might be toxic and may also play a role in Huntington’s disease.

However, follow-up studies are needed to confirm the exact role of all these genes in the process of protein aggregation and to gain more insight into the mechanism(s) of their modifying function.**

### Opposite modifiers 

In our system, knockdown of a number of human genes leads to the opposite effect of knocking down their* C. elegans* homologs. Especially in the functional groups of RNA-synthesis and processing, and protein synthesis, many genes showed a reduction of aggregation in the first screen. The discrepancy between the *C. elegans* and the human results could be caused by the systemic gene-knockdown that is obtained by RNAi in worms compared to knockdown in single human cells. It can be argued that knocking down genes involved in protein synthesis results in a reduction in the overall protein level in cultured cells, leading to lower protein aggregation. On the other hand, knocking down these genes on a systemic level, as in *C. elegans,* could result in stress effects leading to a higher level of protein aggregation in the worm muscle cells. So far, we have not followed up these “negative” modifiers of protein aggregation.**

### Comparison with similar screens in other model systems/organisms

Similar to the *C. elegans* screen on which this study was based [8], others have searched for modifiers of protein aggregation in general, or mutant huntingtin aggregation more specifically. An RNAi-screen in *Drosophila melanogaster *identified 21 novel modifiers of expanded huntingtin aggregation [44], but none of these genes overlapped with the 186 genes we identified in the *C. elegans *screen. However, some genes function in similar pathways, for example *highwire*, a gene functioning in the ubiquitin-proteasome system. The small overlap between the results of the two studies can be explained by many factors: 1) the *Drosophila *screen was performed in a cell line derived from transgenic flies instead of using the full organism; 2) only half of the fly genome was used for RNAi; 3) modifiers in the fly screen were only identified after three rounds of screening, so it was more stringent than our two rounds; and 4) the screen might have been biased towards identification of suppressors by RNAi (i.e. genes that normally function in promoting aggregation) due to the high aggregation propensity of the cell line.

Another screen in *Drosophila *used genome-wide RNAi in *Drosophila* hematocyte-like S2-cells to search for genes that modify aggregation [45] and identified 126 genes involved in different processes. On comparing the outcome of the first round of screening of this *Drosophila *study to the results from our first set of experiments in human cells, there are 34 overlapping genes. But if we compare the final modifiers of aggregation as identified by both studies, only two genes overlap. These genes are t-complex 1 (TCP-1) and ubiquitin-like modifier activating enzyme 1 (UBA1), and both are known to be involved in protein folding and degradation. However, knockdown of most genes identified in the *Drosophila* study led to a reduction in aggregation, whereas in *C. elegans* knocking down the same genes promoted induction. Further studies are required to understand the cause of this discrepancy.**

*Conclusion*

In sum, we investigated modifiers of mutant huntingtin aggregation in human cultured cells based on a genetic study in a small model organism. We show, similar to other screens, that results from small model organisms can be translated to mammalian disease models. We have identified nine novel genetic modifiers of mutant huntingtin aggregation in human cells. Further studies in human neuronal cells or mouse models need to be performed to clarify the cellular pathways by which these genes influence protein aggregation. Such work might lead to novel therapeutic strategies for Huntington's disease and other protein aggregation disorders. 

## **Acknowledgements**

We would like to thank Gijs van Haaften, Maria van Waarde and Klaas Sjollema for technical assistance and Dineke Verbeek and Jackie Senior for editing. 

This research was funded by a Prinses Beatrix Foundation grant, a Rosalind Franklin Fellowship and an Ubbo Emmius grant to E.A.A.N and a Dutch Brain Foundation grant to E.T.  

*Correspondence should be directed to E.A.A.N: e.a.a.nollen@umcg.nl

### **Competing interests**

The authors have declared that no competing interests exist
